# The Impact of the CX3CL1/CX3CR1 Axis in Neurological Disorders

**DOI:** 10.3390/cells9102277

**Published:** 2020-10-13

**Authors:** Paulina Pawelec, Malgorzata Ziemka-Nalecz, Joanna Sypecka, Teresa Zalewska

**Affiliations:** NeuroRepair Department, Mossakowski Medical Research Centre, Polish Academy of Sciences, 02-106 Warsaw, Poland; ppawelec@imdik.pan.pl (P.P.); mnalecz@imdik.pan.pl (M.Z.-N.); jsypecka@imdik.pan.pl (J.S.)

**Keywords:** fractalkine, CX3CR1, cerebral ischemia, epilepsy, neurodegenerative diseases, Alzheimer’s disease, Parkinson’s disease

## Abstract

Fractalkine (FKN, CX3CL1) is a transmembrane chemokine expressed by neurons in the central nervous system (CNS). CX3CL1 signals through its unique receptor, CX3CR1, that is expressed in microglia. Within the CNS, fractalkine acts as a regulator of microglia activation in response to brain injury or inflammation. During the last decade, there has been a growing interest in the roles that the CX3CL1/CX3CR1 signaling pathway plays in the neuropathology of a diverse array of brain disorders. However, the reported results have proven controversial, indicating that a disruption of the CX3CL1 axis induces a disease-specific microglial response that may have either beneficial or detrimental effects. Therefore, it has become clear that the understanding of neuron-to-glia signals mediated by CX3CL1/CX3CR1 at different stages of diseases could provide new insight into potential therapeutic targets. Hence, the aim of this review is to provide a summary of the literature on the emerging role of CX3CL1 in animal models of some brain disorders.

## 1. Introduction

**Figure 1:**
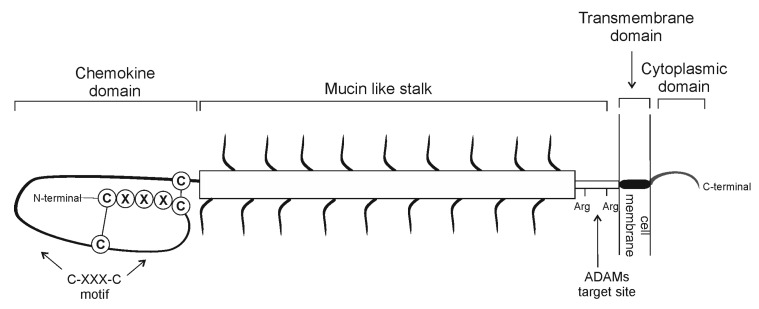
Schematic of the fractalkine (CX3CL1) structure. Fractalkine is expressed as a membrane-bound molecule with a chemokine domain, a heavily glycosylated mucin stalk-like domain attached via a transmembrane domain to the cell surface and a short cytoplasmic domain. Cleavage of CX3CL1 is mediated under physiological and pathological conditions by the proteases ADAM10 and ADAM17.

Fractalkine (FKN, CX3CL1) is a member of the CX3C chemokine family and is constitutively and abundantly expressed in neurons. Fractalkine expression can also be induced in microglia, astrocytes, and vascular endothelial cells [1,2]. CX3CL1 is the only chemokine that is more highly expressed in the CNS than in the periphery [3]. In addition, unlike other chemokines, CX3CL1 can exist either as a static membrane-bound glycoprotein that mediates cell adhesion or as a soluble isoform; this soluble isoform is a product of proteolytic cleavage by disintegrins and metalloproteinases (ADAM10 and ADAM17) and exhibits chemotactic features [4,5,6] (Figure 1). The properties of both the CX3CL1 adhesive and chemotactic isoforms are mediated by a specific G-protein coupled, seven-transmembrane domain receptor (CX3CR1) that is present exclusively on microglial cells. However, these CX3CL1 isoforms may exhibit different affinities and distinctive biological activities associated with specific mediators. The intracellular transmission of signals is mediated by the activation of numerous signaling molecules, including several secondary messengers, transcription factors, signal transducers, and the transcription activator protein AP-1 [7,8,9]. The reciprocal interaction between the microglial chemokine receptor and the neuronal ligand CX3CL1 allows precise and effective communication between neurons and microglial cells and thus plays a key role in coordinating many aspects of brain function. For example, this interaction determines the proper neuronal network, influences synapse maturation and plasticity, regulates cognitive function, and controls immune processes [10,11,12]. Substantial data, in this regard, suggest that the high level of endogenous CX3CL1 expressed in neurons limits CX3CR1 activation on microglia and thus keeps microglia in a quiescent state [13,14].

The altered functionality of the CX3CL1/CX3CR1 pathway in several different pathological states may, on the other hand, promote the activation of microglia and stimulate the release of inflammatory factors [15,16,17,18,19]. The understanding of conveying messages from injured neurons and thus modulating glial cell function has emerged from a number of studies on neuroinflammatory and neurodegenerative disease models during the last decade [20,21,22,23,24]. Recent advances in this field suggest that CX3CL1/CX3CR1 signal disruption is one of the most important elements in the pathogenesis of CNS-related disorders [21,25,26].

**Figure 2:**
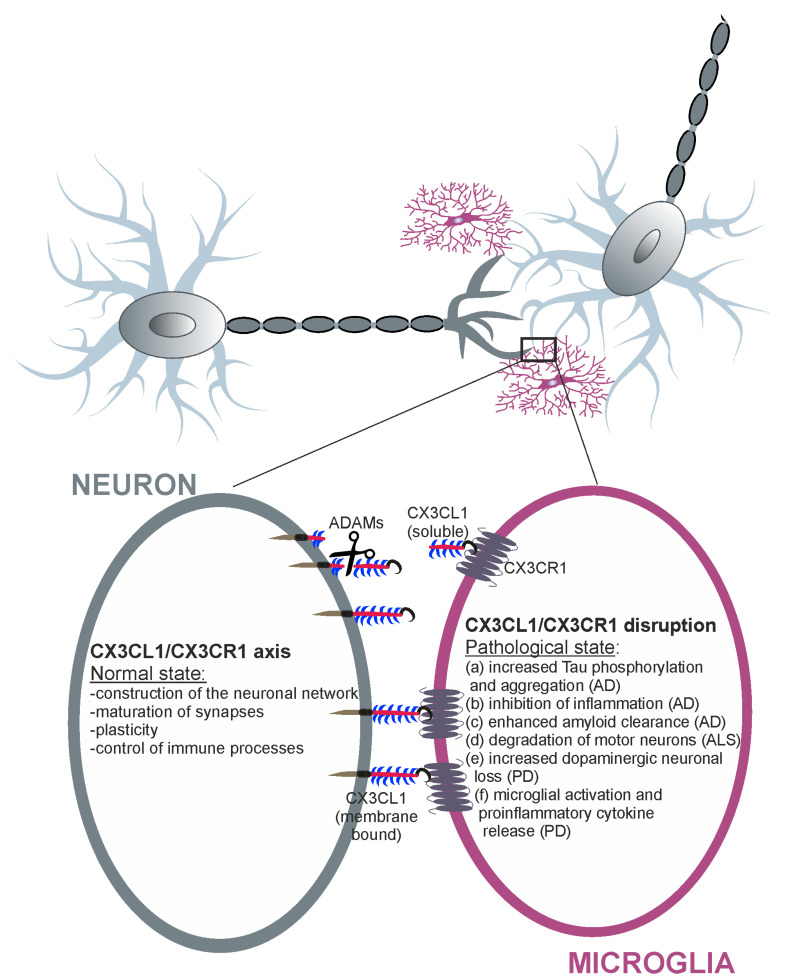
CX3CL1-CX3CR1 signaling between microglia and neurons in physiological and neuropathological conditions. Fractalkine is a membrane-bound or soluble molecule. CX3CL1 bound to the membrane is important for cell adhesion and acts as an “off” signal for microglia. The cleavage of fractalkine is achieved by the ADAM10 and ADAM17 proteases, and this soluble form of CX3CL1 acts as a chemoattractant for inflammatory cells. Disruption of CX3CL1-CX3CR1 signaling in neuropathological diseases may lead to neuroprotective (b, c) and detrimental (a, d, e, f, g) effects.

The loss of the crosstalk that leads to the activation of microglia differently affects the patterns of brain disorders. This loss of crosstalk may either be beneficial for promoting neuronal survival or cause harm to the surrounding cells depending on disease stage and progression (Figure 2). The specific response, neurotoxic or neuroprotective, most likely depends on the type of destructive factor, the CNS area, and the local concentrations of CX3CL1 and CX3CR1 [27]. During the last decade, new approaches have been developed that target the genes encoding the factors of interest (knockout animals), thus shedding light on the role of the fractalkine/receptor axis in several physiological and pathological processes. In this paper, we review literature updates and summarize the current knowledge about the opposing role of the CX3CL1/CX3CR1 signaling pathway in selected neuropathologies (including ischemia, epilepsy, and neurodegenerative diseases).

## 2. CX3CL1/CX3CR1 Signaling in Cerebral Ischemia

It is now widely recognized that brain ischemia triggers a widespread inflammatory reaction that, in conjunction with excitotoxic and oxidative responses, significantly contributes to ischemia-induced neuronal death. Inflammation driven primarily by activating inflammatory glial cells that reside in the CNS together with infiltrating cells of the peripheral immune system (macrophages, mast cells, and monocytes) produces several proinflammatory factors (cytokines, chemokines, NOS, reactive oxygen species, excitatory amino acids and death receptor agonists), which lead to a disruption of cellular homeostasis and structural damage of brain tissue. The recognition of inflammation as the key response to brain injury has stimulated the use of new therapies. A large body of data from cerebral ischemia of different severities consistently shows that at least some neuroprotective effects, including reduced brain damage, can be achieved by decreasing the level of microglial activation [28,29]. A broad range of anti-inflammatory drugs that affect the intracellular pathways in microglial cells serve to protect against insults to the central nervous system.

Recently, a chemokine, fractalkine (CX3CL1), and its unique receptor (CX3CR1) were shown to be able to regulate the activation of microglia to maintain these cells in a quiescent state and thus inhibit the release of inflammatory cytokines [14]. A number of studies have demonstrated that treating microglial or mixed glial cultures with the soluble fractalkine isoform suppresses the lipopolysaccharide (LPS)-induced activation of microglial cells and reduces the production of inflammatory factors, such as NO, IL-6, TNFalpha, and IL-1beta [16,30,31]. Since this noteworthy discovery, it has been postulated that there is a strong link between inflammatory processes and fractalkine signaling pathways, and this theory continues to generate considerable interest. In fact, over the past decade, remarkable advances have been made in understanding and elucidating the phenomenon of the immunosuppressive and neuroprotective potency of fractalkine signaling in a number of different experimental models, including focal and global, transient or permanent ischemia. Several independent studies show that interruption of the CX3CL1/CX3CR1 signaling pathway, either by deletion of *cx3cl1* or deficiency in *cx3cr1* (*cx3cr1^GFP/GFP^*), reduced post-ischemic brain injury. The neuroprotective effect was observed based on the decreased neurological deficit, the diminished release of inflammatory markers (IL-1beta and TNF-alpha), and the ameliorated neuronal death [20,23,24,32,33]. The list of neuroprotective effects also includes the suppression of CNS microglia proliferation, abrogation of macrophage recruitment from the periphery, and promotion of angiogenesis and tissue repair [32,34].

Importantly, there is also evidence that fractalkine reduces microglial activation, maintaining these cells in an “off” state, and inhibits inflammatory cytokines, thereby contributing to its protective activity. According to this statement, the intracerebroventricular administration of exogenous CX3CL1 results in a long-lasting neuroprotective effect against cerebral ischemia in rodents [24,35]. Importantly, exogenous fractalkine participates in angiogenesis in rats subjected to focal ischemia (MCAO). Fractalkine stimulates endothelial cells in the ischemic penumbra to proliferate and migrate, leading to enhanced blood vessel density, which correlates with better functional recovery after ischemic stroke [20,36,37,38]. The mechanism considered above produced a positive effect that may involve inhibition of the caspase 3 activity and pPARP cleavage induced by ischemia [24].

However, although an attempt was made to modulate the immune system of the brain during the acute phase after experimental global cerebral ischemia, fractalkine signaling induced completely different effects. Using small interfering RNA (siRNA), researchers observed that inhibiting the function of CX3CR1 exacerbated the ischemia-induced chronic increase in microglia activation, enhanced the expression of IL-1beta, and worsened the ischemia-induced chronic impairment of cognition [39]. However, there was no significant effect on ischemia-induced neurodegeneration in the hippocampus. Most likely, the regional heterogeneity of microglial cells may contribute to varying sensitivities to the same pathological signal [40]. This fact remains in disagreement with a previous report of a focal ischemia model, in which CX3CR1 deficiency decreased cortical degeneration [20,23,24,32]. These observations may suggest that the protection afforded by this chemokine in temporary occlusion models is more pronounced than that offered in permanent global ischemia models. The contradictory results described above may have occurred due to different experimental models, degrees of injury, and timing of the activation of specific environmental signals, including effector molecules. Furthermore, it is not possible to compare the expression levels of fractalkine/CX3CR1 after insults, as these levels were measured at different times, namely, over a period of 1 week in the acute phase of recovery vs. a period of 1 month after global ischemia. This fact may determine the functional state of activated microglia during the acute and/or late response to ischemic challenge. Indeed, it has previously been reported that microglia produce both beneficial and detrimental effects during cerebral ischemia, which indicates highly complex microglial acute/late function in damage formation, inflammation, and tissue repair.

## 3. CX3CL1/CXCR1 Signaling in Epilepsy

Epilepsy is a chronic neurological disorder characterized by an enduring predisposition to unprovoked seizures. The clinical manifestation of epilepsy includes sudden and transitory abnormal episodes of motor, sensory, autonomic, or psychic origin. Continuous seizure activity may lead to neuronal cell death [41,42].

Seizure episodes are a result of abnormal hypersynchrony of neuronal activities in the brain that are caused by an imbalance of excitatory and inhibitory transmissions [43,44,45]. Recently, some neuroinflammatory processes within the brain, including a predominant role of microglia, have been considered to be the key elements that contribute to seizure reoccurrence and precipitation in both epileptic patients and animal models [46,47,48,49,50,51]. Indeed, it has been reported that mesial temporal lobe epilepsy (MTLE), one of the most common forms of focal epilepsy, is associated with pathology in the hippocampus characterized by acute inflammatory responses, activated parenchymal microglial cells, neurodegeneration, aberrant synaptic reorganization, and increased adult neurogenesis [52,53,54,55,56,57,58]. It has been suggested that microglia, through communication with neuronal elements mediated by the CX3CL1/CX3CR1 axis, monitor and alter synaptic activity under epileptic conditions [59,60,61].

However, only a few studies have shown the role of fractalkine/CX3CR1 axis signaling in the pathogenesis of epilepsy and the accompanying cell death [51,62,63]. These studies have shown increased expression of fractalkine and CX3CR1 in surgically resected brain samples collected from MTLE patients and experimental animal models [63,64]. Blocking the axis with the anti-CX3CR1 antibody diminishes electrical epileptic seizure-induced microglial activation, neurodegeneration, and neuroblast formation in the adult rat hippocampus. Ablation of CX3CR1 receptors in mice leads to reduced epilepsy induced microglial activation, as observed by an increased number of ramified/surveying microglia and a reduced percentage of phagocytic microglial cells, in the hippocampi of the DG. The reduction in microglial cells and the decreased number of degenerated neurons was also described after infusion of a CX3CR1 antibody into the pilocarpine-induced model of epilepsy [61]. An analysis of fractalkine expression showed upregulation of this protein in a rat model and in neocortexes of patients with temporal lobe epilepsy (TLE). Elevated levels of CX3CL1 were also found in the serum and CSF. Unfortunately, most patients who provided brain specimens were unwilling to provide the samples [63]. The observed reduction in the number of neurons after epilepsy is consistent with previous studies [65,66,67]. Therefore, a deficiency in fractalkine signaling is associated with many types of seizures [50].

Recently, Roseti et al. showed that CX3CL1 is responsible for positively regulating the function of the GABA A receptor from human TLE brain tissue when it was expressed in *Xenopus* oocytes. This effect was mediated by reducing the rundown current of the GABA A receptor. Most likely, this receptor was of epileptogenic origin, as it was absent in the nonepileptogenic control tissue [64]. These data may suggest that the GABAergic system is significantly modulated by CX3CL1 released in epileptic foci. This indicates that the CX3CL1/CX3CR1 neuroglial communication axis is a modulator of potentially neuroprotective microglia-neuron physical interactions when neurons tend to be hyperactive. This finding may represent an important challenge for the prevention of epileptogenesis, and the identification of new immunomodulatory compounds remains of utmost importance [64].

## 4. CX3CL1/CX3CR1 Signaling in Neurodegenerative Diseases

Neurodegenerative diseases are a heterogeneous group of disorders that increase in incidence as the population ages. These disorders are characterized by a progressive decline in cognitive function and memory formation, which correlate with reduced neurogenesis and deficits in LTP maintenance in elderly people [68]. Increasing brain and spinal cord damage gradually impairs the function of the central and peripheral nervous systems. This increasing damage finally leads to mental retardation and motor and behavioral problems. The most common neurodegenerative diseases include Alzheimer’s disease (AD), Parkinson’s disease (PD), and amyotrophic lateral sclerosis (ALS). Despite advancing knowledge of the biochemical and molecular processes involved in the pathogenesis of neurological impairments, clinical approaches have failed to prevent the progression of the characteristic symptoms. An important issue regarding neurodegenerative diseases is the identification of appropriate drug targets. Contemporary research suggests that age-dependent neuroinflammatory processes are important contributing factors in most neurological disorders, and while they may not be considered the primary causes, they may contribute to the symptomatic phase [69]. Recently, the signaling pathway mediated by fractalkine CX3CL1 and its receptor CX3CR1 has received considerable attention as an important mediator of inflammatory responses in several neurological disorders [70]. Indeed, several studies have shown that impaired CX3CL1/CX3CR1 axis signaling is accompanied by abnormal microglial activation in various animal models of central nervous system diseases [71,72,73,74,75,76]. Two genetic variants of the *CX3CR1* gene, namely, V249I and T280M, have been described to affect CX3CR1 protein activity [77,78]. According to the reported data, both variants have been associated with several inflammatory and degenerative human conditions, including age-related macular degeneration [79,80], Crohn’s disease [81], AIDS [82], MS [83], ALS, and AD [84,85]. However, to confirm the relevance of these polymorphic variants of CX3CR1, these studies require replication.

Furthermore, fractalkine, the ligand of the CX3CR1 receptor, appears to be protective in some contexts, whereas it contributes to neuronal damage in other contexts. Comparison of the soluble or membrane-bound forms of fractalkine showed that overexpression of soluble fractalkine in knock-out mice has the ability to restore neurogenesis and LTP, whereas membrane-bound CX3CL1 does not have such beneficial effects [86]. This finding might lead to the design of an effective strategy to ameliorate symptoms accompanying many diseases. Our present review summarizes the current data related to the role of the CX3CL1/CX3CR1 signaling pathways in animal models of selected neurodegenerative diseases and their potential use in the clinic.

### 4.1. Fractalkine/CX3CR1 Signaling in Alzheimer’s Disease

Alzheimer’s disease (AD) is one of the most common neurodegenerative disorders and the fifth leading cause of death for individuals aged 65 or older. AD accounts for approximately 80% of all cases of dementia due to progressive cognitive impairment and decreased memory formation associated with neuronal dysfunction [68]. AD is also characterized by noncognitive symptoms such as delusions, agitation, and changes in mood and personality [87].

The two pathological hallmarks of AD are extracellular senile neuritic plaques, of which amyloid beta is the principal component [88], and an intraneuronal accumulation of hyperphosphorylated microtubules associated with the protein Tau, which are known as neurofibrillary tangles. The aggregation of amyloid-β (Aβ) fragments (peptides 40–42) that accumulate to form oligomers induces neurotoxic effects that lead to the neural synaptic and cognitive degradation seen in AD [89]. Intracellular neurofibrillary tangles, as the second hallmark of AD pathology, are involved in the dispersion of microtubules and contribute to the progression of the disease [90].

A body of recently collected evidence indicates that the fractalkine ligand and its microglial receptor (CX3CL1/CX3CR1) affect Aβ and Tau pathologies by regulating microglial movement and recruiting monocytes into the brain. For research purposes, different genetic models (hTau, APP/PS-1, and CRND8) exhibiting specific aspects of the AD disorder were used [91,92,93]. By frequent observation of the brains of both humans and animals with AD, it was concluded that amyloid plaques are surrounded by activated, phagocytic microglia. The fact that microglia proliferate faster and cluster around fibrillar amyloid plaques is probably due to dysregulated fractalkine/CX3CR1 signaling by deletion of the CX3CR1 receptor [25,92,93].

As a result, the total levels of Aβ (particularly Aβ 40 and 42) [93] and the number of amyloid plaques were reduced, most likely by arginase-1-expressing microglia and enhanced microglial selective phagocytic ability [94]. A similar effect occurred when the CX3CL1 was absent. The absence of CX3CL1 reduced Aβ deposition and induced microglial overactivation and cytokine release (IL-6 and IL-1α) in transgenic mice (APP/PS-1) [95]. With disease progression, microglia become less efficient in clearing fibrillar Aβ and heightened activation may help increase microglial engulfment of fibrillar amyloid. [96]. Altogether, it could be assumed that microglia can regulate the levels and deposition of Aβ in the brain. Notably, despite the increased density of microglia, there was no effect of CX3CR1 deletion on the degree of neuronal and synaptic damage surrounding the plaques [93]. In addition, Fuhrmann et al. observed that the absence of CX3CR1 prevented neuronal loss in 3xTg-AD mice and that this event was independent of Aβ accumulation [97]. Nevertheless, several subsequent studies indicated that disrupting the CX3CL1 signaling pathways by knocking out the CX3CR1 receptor on microglia is beneficial in models of amyloid deposition [92,93]. Therefore, it is postulated that the net effect of the absence of CX3CR1 may likely depend on a particular pathological condition [98].

Conversely, a follow-up study surprisingly revealed that ablation of the fractalkine receptor resulted in enhanced Tau pathology, as demonstrated by increased Tau phosphorylation and aggregation, which correlates with worsened behavioral and cognitive deficits [91,99]. Reactive microglia are also involved in enhancing and spreading Tau pathology, which induces neurobrilary tangles (NFT) [100]. Thus, it is likely that the neuroprotective role of the CX3CR1 receptor is not directly associated with amyloid beta but with Tau. This hypothesis may be supported by evidence of substantially improved health in mice affected by Tau pathology after overexpression of soluble fractalkine [25].

In addition, two variants of the *CX3CR1* gene, namely, V249I and T280M, could represent new factors that regulate the onset and progression of pathology in patients with AD. There is data described association of variant CX3CR1-V249I with neurofibrillary pathology. The analysis provides further evidence of the involvement of the CX3CR1 pathway in the pathogenesis of AD [85].

Current evidence has shown that interruption of CX3CL1/CX3CR1 signaling affects the neuron–microglia interaction in AD pathology and it may trigger beneficial or detrimental effects. The beneficial effect is expressed by inhibition of inflammation and amyloid clearance, whereas the negative effect is associated with the enhancement of Tau phosphorylation. The discrepancies in the effects caused by the ligand/receptor axis may result from the use of different models, different stages of disease development, and the utilization of different experimental strategies.

Recently, a novel idea that Tau can directly bind to CX3CR1 and then compete with the natural ligand of this receptor was proposed [101]. This competition may lead to the disruption of neuronal–glial communication and thus uncouple microglial activation. Notably, the binding of Tau to the microglial receptor leads to its own internalization by microglia. Little is known about the consequences of these phenomena in brains of patients with AD, and fully understanding these phenomena may help to design a better, more effective treatment.

An important issue regarding AD pathology is the definition the biological functions of the fractalkine isoforms. It is possible that the signals initiated by either the membrane-bound isoform or soluble isoform may play different roles in amyloid beta clearance and Tau phosphorylation. Importantly, studies have confirmed that overexpressing the soluble form reduced the effect of Tau and did not affect amyloid pathology. This remains consistent with the documented increase in Tau pathology in CX3CR1 null mice and the interruption of the fractalkine signaling pathway [102]. Furthermore, using an adeno-associated viral vector to increase the expression of the soluble CX3CL1 isoform in the rTG4510 mouse model of tauopathy led to reductions in the pathology related to both soluble and insoluble phospho-Tau, ameliorated neuronal loss, and reduced microglia activation [102]. Examining this finding further may lead to the conclusion that soluble CX3CL1 is a potential target for preventing Tau-mediated degeneration. In contrast, membrane-bound fractalkine appears to govern Aβ pathology, as was observed in transgenic APP/PS-1 mice. However, the deficiency in signaling mediated by this isoform causes the intraneuronal microtubule-associated protein Tau (MAPT) to accumulate despite the reduction in Aβ [25].

According to a recently published report, other chemokines and their receptors may compensate for deficiency in CX3CR1. One of these studies suggested the involvement of the chemokines CCL2 or CXCL16 [103]. It was found that CCL2 release is able to reduce plaque formation in mice deficient in the receptor CX3CR1 which is specific for CX3CL. This observation could help to develop a therapeutic strategy for AD [104]. Further research studies that focus on the issue described above are needed to confirm this observation.

### 4.2. CX3CL1/CX3CR1 Signaling in Amyotrophic Lateral Sclerosis

Amyotrophic lateral sclerosis (ALS) is a chronic neurodegenerative disease that mainly affects lower (spinal cord and bulbar) and upper (corticospinal) motor neurons (MNs) [105]. The most common symptoms include progressive muscle weakness, paralysis, and death within 5 years after onset of the disease [106]. Although the mechanism precipitating death of motor neurons has not been precisely defined, studies on animal models of ALS and patients with ALS have revealed many alterations, such as synaptic terminal degeneration, glial cell activation, and sustained neuroinflammation. All these processes have been shown to contribute to motor neuron degeneration in ALS [107,108]. However, the primary events leading to pathology are still controversial. To address this question, different transgenic mice overexpressing mutant SOD1, in which glycine at amino acid position 93 is substituted by alanine or glycine at amino acid position 86 is substituted by arginine (G93A or G86R), were used as ALS models. These animals exhibit key clinical features that are strikingly similar to those of human disease [109,110,111].

Recent findings have confirmed that intercellular communication between motor neurons and microglia plays an important role in the pathogenesis of ALS [108,111,112]. The neuron–glia interaction was disrupted even before the onset of ALS symptoms. However, general information about the relevance of CX3CL1 and CX3CR1 in ALS is rather rare, except that disruption of MN-microglia communication as the result of CX3CR1 receptor deficiency in transgenic *SOD1^G93A^* mice accelerates disease progression and exacerbates neuronal death [21]. This observation indicates that the protective role of CX3CR1 signaling has been proven [112,113]. In addition, CX3CR1 was postulated to be a potential gene that regulates the survival and progression of ALS [113]. However, a study performed on a large population of ALS patients showed that neither of the investigated variants, that is, neither V249I nor T280M, was associated with an increased risk of disease.

This finding directly contradicts a study performed on a much smaller population of individuals, where the presence of V249I was associated with shorter survival [114,115]. Thus, it is obvious that larger cohorts of patients should be enrolled in studies to properly determine the effect of gene polymorphisms on ALS.

### 4.3. Fractalkine/CX3CR1 Signaling in Parkinson’s Disease

Parkinson’s disease (PD) is the second most common neurodegenerative disease, affecting 1–2% of the population over the age of 65 [116,117]. The main features of this pathology are progressive motor dysfunction, such as hypokinesia, resting tremors, rigidity, and postural instability. Moreover, nonmotor symptoms, such as olfactory deficits, constipation, sleep behavior disorders, mood disturbances, and dementia, are also observed in PD patients [117,118]. The neuropathological hallmarks of PD are the presence of Lewy bodies, in which alpha-synuclein is the principal component, and the degenerative processes of dopaminergic neurons in the substantia nigra pars compacta (SNpc), which cause depletion of dopamine in striatal projections [119,120]. Notably, Lewy bodies can also be found in neuronal cells in other neurodegenerative diseases, and they are more prominently produced in the SNpc of individuals with PD.

Although the mechanisms that trigger brain degeneration in PD are unknown, several etiological factors are involved in contributing to the disease, and mitochondrial dysfunction, oxidative stress, proteosomal dysfunction, and neuroinflammation thought to be the key components in the pathogenesis of PD [69,121,122,123]. A comparative study showed that the inflammatory factors found in the brains and cerebral spinal fluid extracted from human PD patients post-mortem (including extensive reactive microgliosis, elevated proinflammatory cytokine expression, lymphocyte infiltration, and loss of TH+ cells in the SNpc) are also observed in rodent and non-human primate models of the disease (viral overexpression model, transgenic and neurotoxin – MPTP, MPP+, 6-OHDA models) and cause the onset and progression of PD [124,125].

Increasing evidence has indicated that the chemokine CX3CL1 and its receptor, CX3CR1, play important roles in modulating the inflammatory response in PD and in other neurological disorders [71,76,126,127].

A series of experiments performed on mice deficient in the microglial receptor CX3CR1 showed that dopaminergic neuronal loss was more pronounced after the neurotoxins MPTP or 6-OHDA had been administered [21,76]. Conversely, intact CX3CL1 to CX3CR1 signaling significantly dampens the effect of 6-OHDA. In addition, injection of CX3CL1 was neuroprotective. Exogenous CX3CL1 counteracted neuronal cell death in the striatum and led to a marked reduction in microglia [76]. Similarly, the beneficial effect associated with CX3CL1 overexpression in the alpha-synuclein PD model was noted by Nash et al. [126].

Furthermore, a detailed study by Morganti et al. about the relative contributions of the soluble and uncleaved membrane-bound fractalkine isoforms allowed these authors to discover, through the use of rAAV gene therapy, that only the soluble fractalkine isoform attenuates the neurotoxic effect of MPTP toxin [127]. Importantly, the neuroprotective action of the soluble form of fractalkine was also juxtaposed with the damage caused by the overexpression of alpha-synuclein [126,128]. The beneficial effects of fractalkine were demonstrated by the improved motor coordination, diminished lesion site, reduced microglial activation and proinflammatory cytokine levels and protected dopaminergic neurons in the SNpc. In contrast, the membrane-bound isoform of fractalkine did not demonstrate neuroprotective capabilities in the investigated models [126,127]. Thus, this important finding may provide evidence that proteolytic cleavage could be a potential mechanism for regulating fractalkine activity in vivo. This observation remains consistent with previous data showing that the exogenous truncated CX3CL1 peptide was able to decrease 6-OHDA-induced neurotoxicity in a rat model of PD [76]. However, the key question that has not yet been fully answered is whether native CX3CL1 was present in the examined animal. Furthermore, Lyon et al. stated that the membrane-bound CX3CL1 isoform exhibits anti-inflammatory activities similar to those of its soluble counterpart [31]; however, this statement remains controversial, as the authors did not compare their results with those obtained after inhibition of the constitutive cleavage mechanism. It seems logical to state that such an experimental paradigm could offer an accurate interpretation.

Taken together, the current data were obtained in studies of neurotoxin models with CX3CR1 deficiency and include contradictory evidence about axis signaling and its effects on the progression of disease. Thome et al. reported that in an alpha-synuclein overexpression model of PD (AA2SYN), deficiency of the receptor attenuates inflammation and fails to exacerbate neurodegeneration [129]. Moreover, an in vitro study of primary mouse microglia showed that the absence of CX3CR1 reduced phagocytosis and the uptake of aggregated alpha-synuclein. Thus, these data suggest that CX3CR1 might influence the progression of synucleopathies in the course of disease. It was further confirmed by Castro-Sanchez et al. [71] that dopaminergic degeneration is exacerbated and that proinflammatory marker production is increased in *Cx3cr1*^−/−^ mice. Most likely, the mechanism that governs the microglial response after alpha-synuclein has been administered is different from the mechanism that is specific to toxin-induced PD [130].

Finally, the differences in the fractalkine signaling pathways could be attributed to the natures of the models and the events that trigger the degenerative processes. For example, the absence of the CX3CL1/CX3CR1 axis yields no nigral dopaminergic neuron loss in either intranasal MPTP- or 6-OHDA-treated mice. In contrast, the absence of the CX3CL1/CX3CR1 axis was deleterious after intraperitoneal MPTP infusion [128].

In the field of PD, fractalkine signaling appears to have potent effects on the neuroinflammatory processes in PD models, but these effects are complex and depend on the nature of the initiating events. It is important to better understand the state of fractalkine signaling in human disease in order to develop neuroprotective strategies to delay the onset or progression of the disease.

## 5. Conclusions

Over the past decade, the understanding of the role the fractalkine pathway plays in brain pathology has greatly advanced. Although there is a growing body of literature, which is supported by extensive studies on the role of the CX3CL1/CX3CR1 signaling pathway in brain diseases, the published results are relatively controversial. While disruption of the fractalkine signaling pathway is beneficial in some pathological states (ischemia), it is detrimental in other neurodegenerative diseases (PD). Furthermore, analysis of AD models suggests that deletion of CX3CR1 may lead to both neuroprotective and detrimental effects. In other neurodegenerative diseases, such as ALS, the reported results are rather rare and do not allow us to precisely determine the role of fractalkine signaling. Furthermore, there is also no complete agreement about the role of the fractalkine isoforms in the development of pathological processes. It only seems logical that future research should be focused on understanding the precise mechanism by which disrupted CX3CL1/CX3CR1 signaling affects microglia and leads to either beneficial or deleterious actions. In summary, emerging evidence suggests that the CX3CL1/CX3CR1 axis is an attractive potential therapeutic target due to its ability to control inflammation in the neurological disorders. Therefore, one of the most urgent issues is to unravel the frame windows where the modulation of neuroinflammatory response suits the different stages of the pathologies.

## References

[1] Yoshida H., Imaizumi T., Fujimoto K., Matsuo N., Kimura K., Cui X., Matsumiya T., Tanji K., Shibata T., Tamo W. (2001). Synergistic stimulation, by tumor necrosis factor-alpha and interferon-gamma, of fractalkine expression in human astrocytes. Neurosci. Lett..

[2] Harrison J.K., Jiang Y., Chen S., Xia Y., Maciejewski D., McNamara R.K., Streit W.J., Salafranca M.N., Adhikari S., Thompson D.A. (1998). Role for neuronally derived fractalkine in mediating interactions between neurons and CX3CR1-expressing microglia. Proc. Natl. Acad. Sci. USA.

[3] Bazan J.F., Bacon K.B., Hardiman G., Wang W., Soo K., Rossi D., Greaves D.R., Zlotnik A., Schall T.J. (1997). A new class of membrane-bound chemokine with a CX3C motif. Nature.

[4] Harrison J.K., Fong A.M., Swain P.A., Chen S., Yu Y.R., Salafranca M.N., Greenleaf W.B., Imai T., Patel D.D. (2001). Mutational analysis of the fractalkine chemokine domain. Basic amino acid residues differentially contribute to CX3CR1 binding, signaling, and cell adhesion. J. Biol. Chem..

[5] Chapman G.A., Moores K., Harrison D., Campbell C.A., Stewart B.R., Strijbos P.J. (2000). Fractalkine cleavage from neuronal membranes represents an acute event in the inflammatory response to excitotoxic brain damage. J. Neurosci..

[6] Hundhausen C., Misztela D., Berkhout T.A., Broadway N., Saftig P., Reiss K., Hartmann D., Fahrenholz F., Postina R., Matthews V. (2003). The disintegrin-like metalloproteinase ADAM10 is involved in constitutive cleavage of CX3CL1 (fractalkine) and regulates CX3CL1-mediated cell-cell adhesion. Blood.

[7] Chandrasekar B., Melby P.C., Sarau H.M., Raveendran M., Perla R.P., Marelli-Berg F.M., Dulin N.O., Singh I.S. (2003). Chemokine-cytokine cross-talk. The ELR+ CXC chemokine LIX (CXCL5) amplifies a proinflammatory cytokine response via a phosphatidylinositol 3-kinase-NF-kappa B pathway. J. Biol. Chem..

[8] Lauro C., Catalano M., Di Paolo E., Chece G., de Costanzo I., Trettel F., Limatola C. (2014). Fractalkine/CX3CL1 engages different neuroprotective responses upon selective glutamate receptor overactivation. Front. Cell. Neurosci..

[9] Gan A.-M., Butoi E.D., Manea A., Simion V., Stan D., Parvulescu M.-M., Calin M., Manduteanu I., Simionescu M. (2013). Inflammatory effects of resistin on human smooth muscle cells: Up-regulation of fractalkine and its receptor, CX3CR1 expression by TLR4 and Gi-protein pathways. Cell Tissue Res..

[10] Yamagata M., Sanes J.R., Weiner J.A. (2003). Synaptic adhesion molecules. Curr. Opin. Cell Biol..

[11] Paolicelli R.C., Bolasco G., Pagani F., Maggi L., Scianni M., Panzanelli P., Giustetto M., Ferreira T.A., Guiducci E., Dumas L. (2011). Synaptic pruning by microglia is necessary for normal brain development. Science.

[12] Lauro C., Catalano M., Trettel F., Limatola C. (2015). Fractalkine in the nervous system: Neuroprotective or neurotoxic molecule?. Ann. N. Y. Acad. Sci..

[13] Biber K., Neumann H., Inoue K., Boddeke H.W.G.M. (2007). Neuronal “On” and “Off” signals control microglia. Trends Neurosci..

[14] Ransohoff R.M., Perry V.H. (2009). Microglial physiology: Unique stimuli, specialized responses. Annu. Rev. Immunol..

[15] Zujovic V., Taupin V. (2003). Use of cocultured cell systems to elucidate chemokine-dependent neuronal/microglial interactions: Control of microglial activation. Methods.

[16] Zujovic V., Benavides J., Vigé X., Carter C., Taupin V. (2000). Fractalkine modulates TNF-alpha secretion and neurotoxicity induced by microglial activation. Glia.

[17] Mattison H.A., Nie H., Gao H., Zhou H., Hong J.-S., Zhang J. (2013). Suppressed pro-inflammatory response of microglia in CX3CR1 knockout mice. J. Neuroimmunol..

[18] Lauro C., Catalano M., Trettel F., Mainiero F., Ciotti M.T., Eusebi F., Limatola C. (2006). The chemokine CX3CL1 reduces migration and increases adhesion of neurons with mechanisms dependent on the beta1 integrin subunit. J. Immunol..

[19] Prinz M., Priller J. (2010). Tickets to the brain: Role of CCR2 and CX3CR1 in myeloid cell entry in the CNS. J. Neuroimmunol..

[20] Soriano S.G., Amaravadi L.S., Wang Y.F., Zhou H., Yu G.X., Tonra J.R., Fairchild-Huntress V., Fang Q., Dunmore J.H., Huszar D. (2002). Mice deficient in fractalkine are less susceptible to cerebral ischemia-reperfusion injury. J. Neuroimmunol..

[21] Cardona A.E., Pioro E.P., Sasse M.E., Kostenko V., Cardona S.M., Dijkstra I.M., Huang D., Kidd G., Dombrowski S., Dutta R. (2006). Control of microglial neurotoxicity by the fractalkine receptor. Nat. Neurosci..

[22] Huang D., Shi F.-D., Jung S., Pien G.C., Wang J., Salazar-Mather T.P., He T.T., Weaver J.T., Ljunggren H.-G., Biron C.A. (2006). The neuronal chemokine CX3CL1/fractalkine selectively recruits NK cells that modify experimental autoimmune encephalomyelitis within the central nervous system. FASEB J..

[23] Denes A., Vidyasagar R., Feng J., Narvainen J., McColl B.W., Kauppinen R.A., Allan S.M. (2007). Proliferating resident microglia after focal cerebral ischaemia in mice. J. Cereb. Blood Flow Metab..

[24] Cipriani R., Villa P., Chece G., Lauro C., Paladini A., Micotti E., Perego C., De Simoni M.-G., Fredholm B.B., Eusebi F. (2011). CX3CL1 is neuroprotective in permanent focal cerebral ischemia in rodents. J. Neurosci..

[25] Lee S., Xu G., Jay T.R., Bhatta S., Kim K.-W., Jung S., Landreth G.E., Ransohoff R.M., Lamb B.T. (2014). Opposing effects of membrane-anchored CX3CL1 on amyloid and tau pathologies via the p38 MAPK pathway. J. Neurosci..

[26] Bachstetter A.D., Morganti J.M., Jernberg J., Schlunk A., Mitchell S.H., Brewster K.W., Hudson C.E., Cole M.J., Harrison J.K., Bickford P.C. (2011). Fractalkine and CX 3 CR1 regulate hippocampal neurogenesis in adult and aged rats. Neurobiol. Aging.

[27] Sheridan G.K., Murphy K.J. (2013). Neuron-glia crosstalk in health and disease: Fractalkine and CX3CR1 take centre stage. Open Biol..

[28] Arvin K.L., Han B.H., Du Y., Lin S., Paul S.M., Holtzman D.M. (2002). Minocycline markedly protects the neonatal brain against hypoxic-ischemic injury. Ann. Neurol..

[29] Dommergues M.-A., Plaisant F., Verney C., Gressens P. (2003). Early microglial activation following neonatal excitotoxic brain damage in mice: A potential target for neuroprotection. Neuroscience.

[30] Mizuno T., Kawanokuchi J., Numata K., Suzumura A. (2003). Production and neuroprotective functions of fractalkine in the central nervous system. Brain Res..

[31] Lyons A., Lynch A.M., Downer E.J., Hanley R., O’Sullivan J.B., Smith A., Lynch M.A. (2009). Fractalkine-induced activation of the phosphatidylinositol-3 kinase pathway attentuates microglial activation in vivo and in vitro. J. Neurochem..

[32] Fumagalli S., Perego C., Ortolano F., De Simoni M.-G. (2013). CX3CR1 deficiency induces an early protective inflammatory environment in ischemic mice. Glia.

[33] Tang Z., Gan Y., Liu Q., Yin J.-X., Liu Q., Shi J., Shi F.-D. (2014). CX3CR1 deficiency suppresses activation and neurotoxicity of microglia/macrophage in experimental ischemic stroke. J. Neuroinflamm..

[34] Michelucci A., Heurtaux T., Grandbarbe L., Morga E., Heuschling P. (2009). Characterization of the microglial phenotype under specific pro-inflammatory and anti-inflammatory conditions: Effects of oligomeric and fibrillar amyloid-beta. J. Neuroimmunol..

[35] Qin W., Li Z., Luo S., Wu R., Pei Z., Huang R. (2014). Exogenous fractalkine enhances proliferation of endothelial cells, promotes migration of endothelial progenitor cells and improves neurological deficits in a rat model of ischemic stroke. Neurosci. Lett..

[36] Ergul A., Alhusban A., Fagan S.C. (2012). Angiogenesis: A harmonized target for recovery after stroke. Stroke.

[37] Fan Y., Yang G.-Y. (2007). Therapeutic angiogenesis for brain ischemia: A brief review. J. Neuroimmune Pharmacol..

[38] Wang Z., Tsai L.-K., Munasinghe J., Leng Y., Fessler E.B., Chibane F., Leeds P., Chuang D.-M. (2012). Chronic valproate treatment enhances postischemic angiogenesis and promotes functional recovery in a rat model of ischemic stroke. Stroke.

[39] Briones T.L., Woods J., Wadowska M. (2014). Chronic neuroinflammation and cognitive impairment following transient global cerebral ischemia: Role of fractalkine/CX3CR1 signaling. J. Neuroinflamm..

[40] Stratoulias V., Venero J.L., Tremblay M.-È., Joseph B. (2019). Microglial subtypes: Diversity within the microglial community. EMBO J..

[41] Fujikawa D.G. (1995). Neuroprotective effect of ketamine administered after status epilepticus onset. Epilepsia.

[42] Rice A.C., DeLorenzo R.J. (1998). NMDA receptor activation during status epilepticus is required for the development of epilepsy. Brain Res..

[43] Fisher R.S., van Emde Boas W., Blume W., Elger C., Genton P., Lee P., Engel J. (2005). Epileptic seizures and epilepsy: Definitions proposed by the International League Against Epilepsy (ILAE) and the International Bureau for Epilepsy (IBE). Epilepsia.

[44] Dalby N.O., Mody I. (2001). The process of epileptogenesis: A pathophysiological approach. Curr. Opin. Neurol..

[45] Sharma A.K., Reams R.Y., Jordan W.H., Miller M.A., Thacker H.L., Snyder P.W. (2007). Mesial temporal lobe epilepsy: Pathogenesis, induced rodent models and lesions. Toxicol. Pathol..

[46] De Vries H.E., Kooij G., Frenkel D., Georgopoulos S., Monsonego A., Janigro D. (2012). Inflammatory events at blood-brain barrier in neuroinflammatory and neurodegenerative disorders: Implications for clinical disease. Epilepsia.

[47] Devinsky O., Vezzani A., Najjar S., De Lanerolle N.C., Rogawski M.A. (2013). Glia and epilepsy: Excitability and inflammation. Trends Neurosci..

[48] Vezzani A., Aronica E., Mazarati A., Pittman Q.J. (2013). Epilepsy and brain inflammation. Exp. Neurol..

[49] Aronica E., Bauer S., Bozzi Y., Caleo M., Dingledine R., Gorter J.A., Henshall D.C., Kaufer D., Koh S., Löscher W. (2017). Neuroinflammatory targets and treatments for epilepsy validated in experimental models. Epilepsia.

[50] Eyo U.B., Peng J., Murugan M., Mo M., Lalani A., Xie P., Xu P., Margolis D.J., Wu L.-J. (2016). Regulation of Physical Microglia-Neuron Interactions by Fractalkine Signaling after Status Epilepticus. eNeuro.

[51] Cerri C., Caleo M., Bozzi Y. (2017). Chemokines as new inflammatory players in the pathogenesis of epilepsy. Epilepsy Res..

[52] Bengzon J., Kokaia Z., Elmér E., Nanobashvili A., Kokaia M., Lindvall O. (1997). Apoptosis and proliferation of dentate gyrus neurons after single and intermittent limbic seizures. Proc. Natl. Acad. Sci. USA.

[53] Parent J.M., Yu T.W., Leibowitz R.T., Geschwind D.H., Sloviter R.S., Lowenstein D.H. (1997). Dentate granule cell neurogenesis is increased by seizures and contributes to aberrant network reorganization in the adult rat hippocampus. J. Neurosci..

[54] De Simoni M.G., Perego C., Ravizza T., Moneta D., Conti M., Marchesi F., De Luigi A., Garattini S., Vezzani A. (2000). Inflammatory cytokines and related genes are induced in the rat hippocampus by limbic status epilepticus. Eur. J. Neurosci..

[55] Scharfman H., Pedley T. (2007). Temporal Lobe Epilepsy. Neurobiology of Disease.

[56] Ravizza T., Gagliardi B., Noé F., Boer K., Aronica E., Vezzani A. (2008). Innate and adaptive immunity during epileptogenesis and spontaneous seizures: Evidence from experimental models and human temporal lobe epilepsy. Neurobiol. Dis..

[57] Lu Y., Xue T., Yuan J., Li Y., Wu Y., Xi Z., Xiao Z., Chen Y., Wang X. (2009). Increased expression of TGFbeta type I receptor in brain tissues of patients with temporal lobe epilepsy. Clin. Sci..

[58] Ali I., Chugh D., Ekdahl C.T. (2015). Role of fractalkine-CX3CR1 pathway in seizure-induced microglial activation, neurodegeneration, and neuroblast production in the adult rat brain. Neurobiol. Dis..

[59] Wake H., Moorhouse A.J., Jinno S., Kohsaka S., Nabekura J. (2009). Resting microglia directly monitor the functional state of synapses in vivo and determine the fate of ischemic terminals. J. Neurosci..

[60] Tremblay M.-È., Lowery R.L., Majewska A.K. (2010). Microglial interactions with synapses are modulated by visual experience. PLoS Biol..

[61] Kato G., Inada H., Wake H., Akiyoshi R., Miyamoto A., Eto K., Ishikawa T., Moorhouse A.J., Strassman A.M., Nabekura J. (2016). Microglial Contact Prevents Excess Depolarization and Rescues Neurons from Excitotoxicity. eNeuro.

[62] Yeo S.-I., Kim J.-E., Ryu H.J., Seo C.H., Lee B.C., Choi I.-G., Kim D.-S., Kang T.-C. (2011). The roles of fractalkine/CX3CR1 system in neuronal death following pilocarpine-induced status epilepticus. J. Neuroimmunol..

[63] Xu Y., Zeng K., Han Y., Wang L., Chen D., Xi Z., Wang H., Wang X., Chen G. (2012). Altered expression of CX3CL1 in patients with epilepsy and in a rat model. Am. J. Pathol..

[64] Roseti C., Fucile S., Lauro C., Martinello K., Bertollini C., Esposito V., Mascia A., Catalano M., Aronica E., Limatola C. (2013). Fractalkine/CX3CL1 modulates GABAA currents in human temporal lobe epilepsy. Epilepsia.

[65] Li J.-M., Wang X.-F., Xi Z.-Q., Gong Y., Liu F.-Y., Sun J.-J., Wu Y., Luan G.-M., Wang Y.-P., Li Y.-L. (2006). Decreased expression of thyroid receptor-associated protein 220 in temporal lobe tissue of patients with refractory epilepsy. Biochem. Biophys. Res. Commun..

[66] Wang L., Pan Y., Chen D., Xiao Z., Xi Z., Xiao F., Wang X. (2010). Tetranectin is a potential biomarker in cerebrospinal fluid and serum of patients with epilepsy. Clin. Chim. Acta.

[67] Xi Z.-Q., Sun J.-J., Wang X.-F., Li M.-W., Liu X.-Z., Wang L.-Y., Zhu X., Xiao F., Li J.-M., Gong Y. (2007). HSPBAP1 is found extensively in the anterior temporal neocortex of patients with intractable epilepsy. Synapse.

[68] Minati L., Edginton T., Bruzzone M.G., Giaccone G. (2009). Current concepts in Alzheimer’s disease: A multidisciplinary review. Am. J. Alzheimers Dis. Other Dement..

[69] Hirsch E.C., Hunot S. (2009). Neuroinflammation in Parkinson’s disease: A target for neuroprotection?. Lancet Neurol..

[70] Luo P., Chu S.-F., Zhang Z., Xia C.-Y., Chen N.-H. (2019). Fractalkine/CX3CR1 is involved in the cross-talk between neuron and glia in neurological diseases. Brain Res. Bull..

[71] Castro-Sánchez S., García-Yagüe Á.J., López-Royo T., Casarejos M., Lanciego J.L., Lastres-Becker I. (2018). Cx3cr1-deficiency exacerbates alpha-synuclein-A53T induced neuroinflammation and neurodegeneration in a mouse model of Parkinson’s disease. Glia.

[72] Zhang L., Xu J., Gao J., Wu Y., Yin M., Zhao W. (2018). CD200-, CX3CL1-, and TREM2-mediated neuron-microglia interactions and their involvements in Alzheimer’s disease. Rev. Neurosci..

[73] Zujovic V., Schussler N., Jourdain D., Duverger D., Taupin V. (2001). In vivo neutralization of endogenous brain fractalkine increases hippocampal TNFalpha and 8-isoprostane production induced by intracerebroventricular injection of LPS. J. Neuroimmunol..

[74] Mizutani N., Sakurai T., Shibata T., Uchida K., Fujita J., Kawashima R., Kawamura Y.I., Toyama-Sorimachi N., Imai T., Dohi T. (2007). Dose-dependent differential regulation of cytokine secretion from macrophages by fractalkine. J. Immunol..

[75] Wynne A.M., Henry C.J., Huang Y., Cleland A., Godbout J.P. (2010). Protracted downregulation of CX3CR1 on microglia of aged mice after lipopolysaccharide challenge. Brain Behav. Immun..

[76] Pabon M.M., Bachstetter A.D., Hudson C.E., Gemma C., Bickford P.C. (2011). CX3CL1 reduces neurotoxicity and microglial activation in a rat model of Parkinson’s disease. J. Neuroinflamm..

[77] McDermott D.H., Fong A.M., Yang Q., Sechler J.M., Cupples L.A., Merrell M.N., Wilson P.W.F., D’Agostino R.B., O’Donnell C.J., Patel D.D. (2003). Chemokine receptor mutant CX3CR1-M280 has impaired adhesive function and correlates with protection from cardiovascular disease in humans. J. Clin. Investig..

[78] Daoudi M., Lavergne E., Garin A., Tarantino N., Debré P., Pincet F., Combadière C., Deterre P. (2004). Enhanced adhesive capacities of the naturally occurring Ile249-Met280 variant of the chemokine receptor CX3CR1. J. Biol. Chem..

[79] Zhang R., Wang L.-Y., Wang Y.-F., Wu C.-R., Lei C.-L., Wang M.-X., Ma L. (2015). Associations Between the T280M and V249I SNPs in CX3CR1 and the Risk of Age-Related Macular Degeneration. Investig. Ophthalmol. Vis. Sci..

[80] Ma B., Dang G., Yang S., Duan L., Zhang Y. (2015). CX3CR1 polymorphisms and the risk of age-related macular degeneration. Int. J. Clin. Exp. Pathol..

[81] Brand S., Hofbauer K., Dambacher J., Schnitzler F., Staudinger T., Pfennig S., Seiderer J., Tillack C., Konrad A., Göke B. (2006). Increased expression of the chemokine fractalkine in Crohn’s disease and association of the fractalkine receptor T280M polymorphism with a fibrostenosing disease Phenotype. Am. J. Gastroenterol..

[82] Faure S., Meyer L., Costagliola D., Vaneensberghe C., Genin E., Autran B., Delfraissy J.F., McDermott D.H., Murphy P.M., Debré P. (2000). Rapid progression to AIDS in HIV+ individuals with a structural variant of the chemokine receptor CX3CR1. Science.

[83] Arli B., Irkec C., Menevse S., Yilmaz A., Alp E. (2013). Fractalkine gene receptor polymorphism in patients with multiple sclerosis. Int. J. Neurosci..

[84] Lopez-Lopez A., Gamez J., Syriani E., Morales M., Salvado M., Rodríguez M.J., Mahy N., Vidal-Taboada J.M. (2014). CX3CR1 is a modifying gene of survival and progression in amyotrophic lateral sclerosis. PLoS ONE.

[85] López-López A., Gelpi E., Lopategui D.M., Vidal-Taboada J.M. (2018). Association of the CX3CR1-V249I Variant with Neurofibrillary Pathology Progression in Late-Onset Alzheimer’s Disease. Mol. Neurobiol..

[86] Winter A.N., Subbarayan M.S., Grimmig B., Weesner J.A., Moss L., Peters M., Weeber E., Nash K., Bickford P.C. (2020). Two forms of CX3CL1 display differential activity and rescue cognitive deficits in CX3CL1 knockout mice. J. Neuroinflamm..

[87] Epelbaum S., Genthon R., Cavedo E., Habert M.O., Lamari F., Gagliardi G., Lista S., Teichmann M., Bakardjian H., Hampel H. (2017). Preclinical Alzheimer’s disease: A systematic review of the cohorts underlying the concept. Alzheimers Dement..

[88] Alzheimer A., Stelzmann R.A., Schnitzlein H.N., Murtagh F.R. (1995). An English translation of Alzheimer’s 1907 paper, “Uber eine eigenartige Erkankung der Hirnrinde”. Clin. Anat..

[89] Hardy J., Selkoe D.J. (2002). The amyloid hypothesis of Alzheimer’s disease: Progress and problems on the road to therapeutics. Science.

[90] Irwin D.J., Cohen T.J., Grossman M., Arnold S.E., Xie S.X., Lee V.M.-Y., Trojanowski J.Q. (2012). Acetylated tau, a novel pathological signature in Alzheimer’s disease and other tauopathies. Brain.

[91] Bhaskar K., Konerth M., Kokiko-Cochran O.N., Cardona A., Ransohoff R.M., Lamb B.T. (2010). Regulation of tau pathology by the microglial fractalkine receptor. Neuron.

[92] Lee S., Varvel N.H., Konerth M.E., Xu G., Cardona A.E., Ransohoff R.M., Lamb B.T. (2010). CX3CR1 deficiency alters microglial activation and reduces beta-amyloid deposition in two Alzheimer’s disease mouse models. Am. J. Pathol..

[93] Liu Z., Condello C., Schain A., Harb R., Grutzendler J. (2010). CX3CR1 in microglia regulates brain amyloid deposition through selective protofibrillar amyloid-β phagocytosis. J. Neurosci..

[94] Cherry J.D., Olschowka J.A., O’Banion M.K. (2015). Arginase 1+ microglia reduce Aβ plaque deposition during IL-1β-dependent neuroinflammation. J. Neuroinflamm..

[95] Merino J.J., Muñetón-Gómez V., Alvárez M.-I., Toledano-Díaz A. (2016). Effects of CX3CR1 and Fractalkine Chemokines in Amyloid Beta Clearance and p-Tau Accumulation in Alzheimer’s Disease (AD) Rodent Models: Is Fractalkine a Systemic Biomarker for AD?. Curr. Alzheimer Res..

[96] DiCarlo G., Wilcock D., Henderson D., Gordon M., Morgan D. (2001). Intrahippocampal LPS injections reduce Abeta load in APP+PS1 transgenic mice. Neurobiol. Aging.

[97] Fuhrmann M., Bittner T., Jung C.K.E., Burgold S., Page R.M., Mitteregger G., Haass C., LaFerla F.M., Kretzschmar H., Herms J. (2010). Microglial Cx3cr1 knockout prevents neuron loss in a mouse model of Alzheimer’s disease. Nat. Neurosci..

[98] Hanisch U.-K., Kettenmann H. (2007). Microglia: Active sensor and versatile effector cells in the normal and pathologic brain. Nat. Neurosci..

[99] Cho S.-H., Sun B., Zhou Y., Kauppinen T.M., Halabisky B., Wes P., Ransohoff R.M., Gan L. (2011). CX3CR1 protein signaling modulates microglial activation and protects against plaque-independent cognitive deficits in a mouse model of Alzheimer disease. J. Biol. Chem..

[100] Maphis N., Xu G., Kokiko-Cochran O.N., Jiang S., Cardona A., Ransohoff R.M., Lamb B.T., Bhaskar K. (2015). Reactive microglia drive tau pathology and contribute to the spreading of pathological tau in the brain. Brain.

[101] Bolós M., Llorens-Martín M., Perea J.R., Jurado-Arjona J., Rábano A., Hernández F., Avila J. (2017). Absence of CX3CR1 impairs the internalization of Tau by microglia. Mol. Neurodegener..

[102] Nash K.R., Lee D.C., Hunt J.B., Morganti J.M., Selenica M.-L., Moran P., Reid P., Brownlow M., Guang-Yu Yang C., Savalia M. (2013). Fractalkine overexpression suppresses tau pathology in a mouse model of tauopathy. Neurobiol. Aging.

[103] Rosito M., Lauro C., Chece G., Porzia A., Monaco L., Mainiero F., Catalano M., Limatola C., Trettel F. (2014). Trasmembrane chemokines CX3CL1 and CXCL16 drive interplay between neurons, microglia and astrocytes to counteract pMCAO and excitotoxic neuronal death. Front. Cell. Neurosci..

[104] Jaerve A., Müller H.W. (2012). Chemokines in CNS injury and repair. Cell Tissue Res..

[105] Brain L., Walton J. (1969). Brain’s Diseases of the Nervous System.

[106] Rowland L.P., Shneider N.A. (2001). Amyotrophic lateral sclerosis. N. Engl. J. Med..

[107] Turner M.R., Parton M.J., Shaw C.E., Leigh P.N., Al-Chalabi A. (2003). Prolonged survival in motor neuron disease: A descriptive study of the King’s database 1990–2002. J. Neurol. Neurosurg. Psychiatry.

[108] Brites D., Vaz A.R. (2014). Microglia centered pathogenesis in ALS: Insights in cell interconnectivity. Front. Cell. Neurosci..

[109] Bruijn L.I., Becher M.W., Lee M.K., Anderson K.L., Jenkins N.A., Copeland N.G., Sisodia S.S., Rothstein J.D., Borchelt D.R., Price D.L. (1997). ALS-linked SOD1 mutant G85R mediates damage to astrocytes and promotes rapidly progressive disease with SOD1-containing inclusions. Neuron.

[110] Dupuis L., de Tapia M., René F., Lutz-Bucher B., Gordon J.W., Mercken L., Pradier L., Loeffler J.P. (2000). Differential screening of mutated SOD1 transgenic mice reveals early up-regulation of a fast axonal transport component in spinal cord motor neurons. Neurobiol. Dis..

[111] Ripps M.E., Huntley G.W., Hof P.R., Morrison J.H., Gordon J.W. (1995). Transgenic mice expressing an altered murine superoxide dismutase gene provide an animal model of amyotrophic lateral sclerosis. Proc. Natl. Acad. Sci. USA.

[112] Liu C., Hong K., Chen H., Niu Y., Duan W., Liu Y., Ji Y., Deng B., Li Y., Li Z. (2019). Evidence for a protective role of the CX3CL1/CX3CR1 axis in a model of amyotrophic lateral sclerosis. Biol. Chem..

[113] Wolf Y., Yona S., Kim K.-W., Jung S. (2013). Microglia, seen from the CX3CR1 angle. Front. Cell. Neurosci..

[114] Calvo A., Moglia C., Canosa A., Cammarosano S., Ilardi A., Bertuzzo D., Traynor B.J., Brunetti M., Barberis M., Mora G. (2018). Common polymorphisms of chemokine (C-X3-C motif) receptor 1 gene modify amyotrophic lateral sclerosis outcome: A population-based study. Muscle Nerve.

[115] Ghasemi M., Brown R.H. (2018). Genetics of Amyotrophic Lateral Sclerosis. Cold Spring Harb. Perspect. Med..

[116] Hirsch L., Jette N., Frolkis A., Steeves T., Pringsheim T. (2016). The Incidence of Parkinson’s Disease: A Systematic Review and Meta-Analysis. Neuroepidemiology.

[117] Mack J.M., Schamne M.G., Sampaio T.B., Pértile R.A.N., Fernandes P.A.C.M., Markus R.P., Prediger R.D. (2016). Melatoninergic System in Parkinson’s Disease: From Neuroprotection to the Management of Motor and Nonmotor Symptoms. Oxid Med. Cell. Longev..

[118] Chaudhuri K.R., Healy D.G., Schapira A.H.V. (2006). National Institute for Clinical Excellence Non-motor symptoms of Parkinson’s disease: Diagnosis and management. Lancet Neurol..

[119] Gao L., Zhou W., Symmes B., Freed C.R. (2016). Re-Cloning the N27 Dopamine Cell Line to Improve a Cell Culture Model of Parkinson’s Disease. PLoS ONE.

[120] Sharma S., Taliyan R., Singh S. (2015). Beneficial effects of sodium butyrate in 6-OHDA induced neurotoxicity and behavioral abnormalities: Modulation of histone deacetylase activity. Behav. Brain Res..

[121] McGeer P.L., Itagaki S., Akiyama H., McGeer E.G. (1988). Rate of cell death in parkinsonism indicates active neuropathological process. Ann. Neurol..

[122] Tieu K., Ischiropoulos H., Przedborski S. (2003). Nitric oxide and reactive oxygen species in Parkinson’s disease. IUBMB Life.

[123] Lastres-Becker I. (2017). Role of the Transcription Factor Nrf2 in Parkinson’s Disease: New Insights. J. Alzheimer’s Dis. Parkinsonism.

[124] Allen Reish H.E., Standaert D.G. (2015). Role of α-synuclein in inducing innate and adaptive immunity in Parkinson disease. J. Parkinson’s Dis..

[125] Ramsey C.P., Tansey M.G. (2014). A survey from 2012 of evidence for the role of neuroinflammation in neurotoxin animal models of Parkinson’s disease and potential molecular targets. Exp. Neurol..

[126] Nash K.R., Moran P., Finneran D.J., Hudson C., Robinson J., Morgan D., Bickford P.C. (2015). Fractalkine over expression suppresses α-synuclein-mediated neurodegeneration. Mol. Ther..

[127] Morganti J.M., Nash K.R., Grimmig B.A., Ranjit S., Small B., Bickford P.C., Gemma C. (2012). The soluble isoform of CX3CL1 is necessary for neuroprotection in a mouse model of Parkinson’s disease. J. Neurosci..

[128] Tristão F.S.M., Lazzarini M., Martin S., Amar M., Stühmer W., Kirchhoff F., Gomes L.A.C., Lanfumey L., Prediger R.D., Sepulveda J.E. (2016). CX3CR1 Disruption Differentially Influences Dopaminergic Neuron Degeneration in Parkinsonian Mice Depending on the Neurotoxin and Route of Administration. Neurotox. Res..

[129] Thome A.D., Standaert D.G., Harms A.S. (2015). Fractalkine Signaling Regulates the Inflammatory Response in an α-Synuclein Model of Parkinson Disease. PLoS ONE.

[130] Lastres-Becker I., Innamorato N.G., Jaworski T., Rábano A., Kügler S., Van Leuven F., Cuadrado A. (2014). Fractalkine activates NRF2/NFE2L2 and heme oxygenase 1 to restrain tauopathy-induced microgliosis. Brain.

